# A Case of Sickle Cell Trait Presenting With Stroke

**DOI:** 10.7759/cureus.97304

**Published:** 2025-11-20

**Authors:** Mihiran Thanigasalan

**Affiliations:** 1 Internal Medicine, National Hospital of Sri Lanka, Colombo, LKA

**Keywords:** red cell exchange transfusion, sickle cell disease, sickle cell trait, stroke, vertebral artery occlusion

## Abstract

We present the case of a 41-year-old previously well smoker presenting with acute-onset, left-sided lower motor neuron facial nerve palsy, left trigeminal sensory loss, and cerebellar signs. Imaging with non-contrast CT and MRI of the brain excluded the diagnosis of a cerebellopontine angle tumor and multiple sclerosis, and was concluded as a stroke. As the patient was from a well-identified cluster area in Sri Lanka, the sickling test was done. The sickling test was positive, and we proceeded with high-performance liquid chromatography, which revealed 38.2% of hemoglobin S. The diagnosis of the trait was confirmed with genetic analysis. A CT angiogram of the major branches of the aorta revealed abnormal retrograde flow in the left vertebral artery in the Doppler study. The findings of the CT angiogram were consistent with the total occlusion at the proximal part of the left vertebral artery. Stenosis of the major extracranial arteries is observed in sickle cell disease, but it is rare. This can be attributed to chronic sickle vasculopathy. This phenomenon is not seen in sickle cell trait. Regular screening for sickle cell disease or trait is crucial in young stroke patients originating from endemic regions, even in the absence of anemia. Sickle cell trait may contribute to ischemic stroke, potentially associated with extracranial arteriopathy. Further investigation is warranted to elucidate its role in the pathogenesis of stroke.

## Introduction

Sickle cell disease is a group of hemoglobin disorders resulting from the inheritance of the sickle *β-globin* gene. The resultant hemoglobin is insoluble and polymerizes when exposed to low oxygen tensions. In sickle cell disease, the red cells sickle and may block different areas of microcirculation or large vessels, resulting in infarction of the organs.

Sickle cell trait is considered a benign condition, characterized by the normal appearance of red blood cells in a blood film. Most patients with sickle cell trait usually do not have any symptoms except for hematuria. Rarely, they present with the harmful sequences of sickling in some extreme circumstances. Although documented, it is still not widely appreciated that the sickle cell trait can cause stroke due to the contradictory evidence [1,2].

## Case presentation

A previously well 41-year-old gentleman presented with acute-onset numbness and weakness on the left side of the face, vertigo, and unsteadiness for four days. He did not complain of weakness or numbness in any limbs. There was no history of difficulty in swallowing, speaking, double vision, vision loss, or incontinence. There was no history of headache, early morning vomiting, or fits. There was no history of arthralgia and stiffness of the joints. No oral ulcers, hair loss, or rashes were noted. There was no history of palpitation, chest pain, or documented coronary artery disease. No dry eyes or dry mouth was noted. Further, there was no history of renal impairment or hematuria. There was no history of red eye, deep vein thrombosis, or genital ulcers. He was not on any medications. There were two episodes of loss of consciousness. The first episode occurred nine months ago when he was rafting, and the second occurred four months ago when he was carrying a coffin on his shoulder during a funeral in the hill country. Both episodes were abrupt in onset and lasted only for two minutes, and he gained consciousness completely. There was no family history of diabetes, hypertension, connective tissue disorders, anemia, or young strokes. He had smoked two to three cigarettes per day for the last 10 years. He reported occasional alcohol consumption and no other risk behaviors.

The patient was an averagely built man with a body mass index of 22.4 kg/m². He was neither pale nor plethoric. There was no corneal arcus, oral ulcers, rashes, or tender joints. No eruptive xanthoma or ankle edema was noted. There was left-sided lower motor neuron facial nerve palsy and left-sided trigeminal sensory palsy. No other cranial nerves were involved. He had nystagmus with the fast component toward the left side. There were left-sided cerebellar signs. Tone, power, and reflexes were normal in all four limbs. There was no sensory deficit.

The pulse rate was 72 beats/minute, regular with good volume. All peripheral pulses were felt, and there were no delays. Blood pressure was 130/80 mmHg in both arms. Heart sounds were normal with no murmurs. No carotid or subclavian bruits were noted. There was no organomegaly, masses, or renal bruits. Respiratory examination was unremarkable.

Posterior circulation stroke was considered the first differential diagnosis. As he was young, vasculitis, thrombotic disorders, and hematological disorders were considered. A cerebellopontine angle tumor was considered a second differential diagnosis. But there was no history of headache, and the onset was acute. Multiple sclerosis was also considered, as he did not have atherosclerotic risk factors except for smoking.

There was no anemia or polycythemia, and the other cell lines were within the normal range. Blood sugar and the lipid profile were normal. ECG did not show any arrhythmia. Erythrocyte sedimentation rate and coagulation profile were normal. Young stroke evaluation was performed. A non-contrast CT (NCCT) of the brain performed on admission was normal.

**Figure 1:**
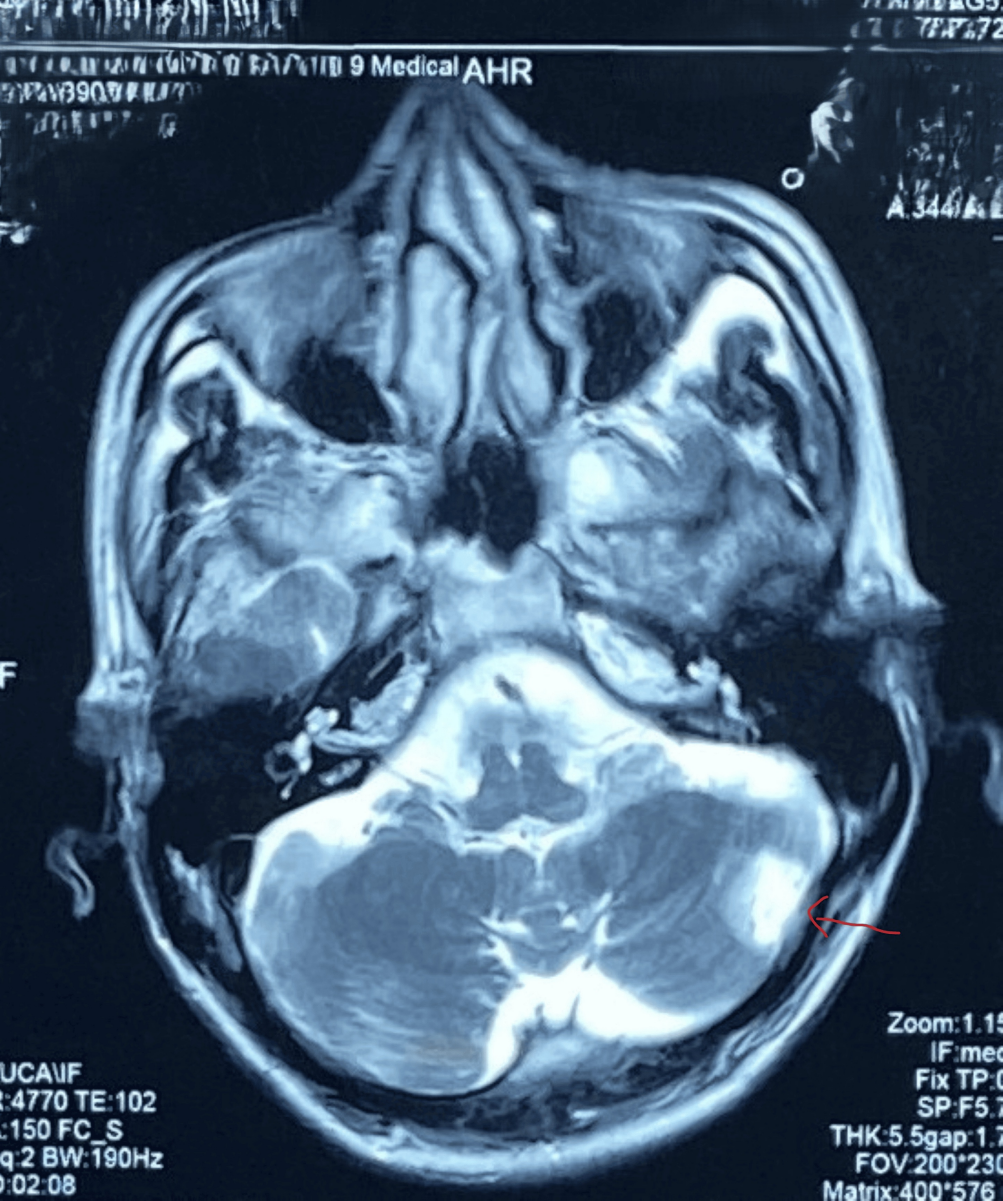
Axial T2-weighted MRI of the brain. The arrow showa a focal area of signal intensity in the left cerebellar hemisphere.

**Figure 2:**
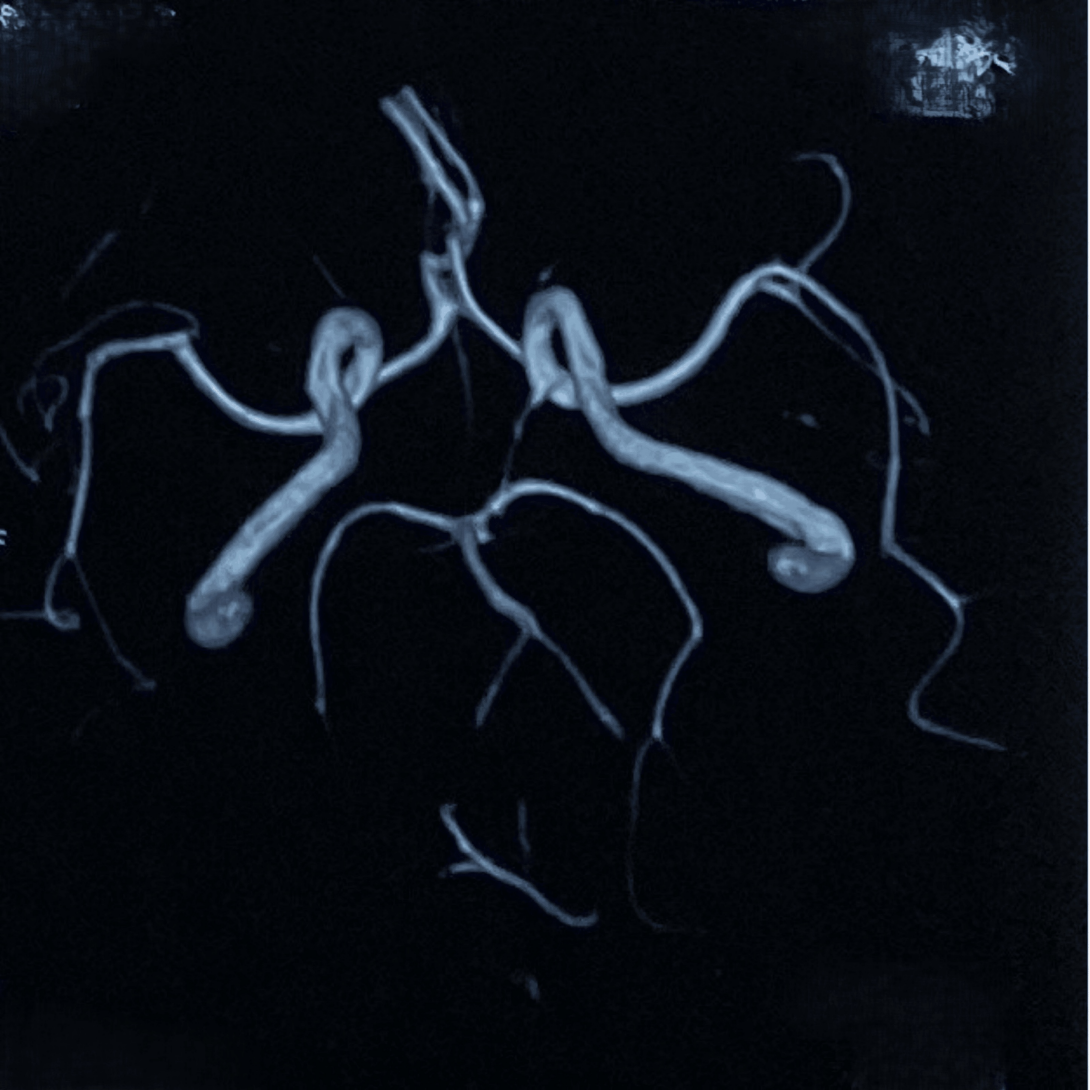
Normal MR angiography of the circle of Willis.

MRI of the brain obtained 72 hours after admission revealed a subacute infarction in the left middle cerebellar peduncle and an old lacunar infarction (Figure 1). The MR angiogram was normal (Figure 2). Given his origin from a well-identified sickle cell cluster area, a sickling test was performed, which was positive [3]. High-performance liquid chromatography showed 38.2% hemoglobin S, confirming sickle cell trait (Table 1). Genetic analysis further validated the diagnosis.

**Table 1:** Investigation findings.

Parameter	Patient’s value	Reference range
White blood cell count	9,110/µL	4,000–11,000/µL
Red blood cell count	4,700,000/µL	4,600,000–6,100,000/µL
Hemoglobin	13.6 g/dL	12–15.5 g/dL
Platelet	217,000/µL	150,000–450,000/µL
International normalized ration	0.86	0.8–1.2
Activated partial thromboplastin time	32 seconds	24–35 seconds
Serum creatinine	75 µmol/L	53–97 µmol/L
Non-contrast CT brain	No hemorrhages and no space-occupying lesions	-
Fasting blood sugar	94 mg/dL	70–99 mg/dL
Lipid profile		
Total cholesterol	190 mg/dL	140–239 mg/dL
Low-density lipoprotein	110 mg/dL	40–159 mg/dL
High-density lipoprotein	55 mg/dL	35–85 mg/dL
Triglycerides	120 mg/dL	10–200 mg/dL
ECG	Normal sinus rhythm	-
Two-dimensional echocardiography	No valvular abnormality, no vegetation, ejection fraction >60%	-
24-hour Holter monitoring	No significant abnormality	-
Erythrocyte sedimentation rate	18 mm/1^st^ hour	<30 mm/1^st^ hour
Carotid and vertebral artery Doppler	Low-velocity retrograde flow of the left-sided vertebral artery. Otherwise normal	-
MRI of the brain	Sub-acute infarction involving the left middle cerebellar peduncle and old lacunar infarction in the left cerebellar hemisphere	-
MR angiography of the brain	Normal	-
Antinuclear antibodies	Negative	-
IgG anti-cardiolipin antibodies	Negative	-
High-performance liquid chromatography		
Hemoglobin A	52%	95–98%
Hemoglobin A_2_	2.7%	2–3.5%
Hemoglobin F	0.9%	0.8–2%
Hemoglobin S	38.2%	0%
HIV 1 and 2 antibody	Negative	-
CT angiogram of the major branches of aorta up to the base of the skull	Total occlusion of the pre-foraminal and proximal part of the foraminal segment of the left vertebral artery. Right side vertebral, subclavian, common carotid, internal, and external carotid arteries were opacified normally	-

**Figure 3:**
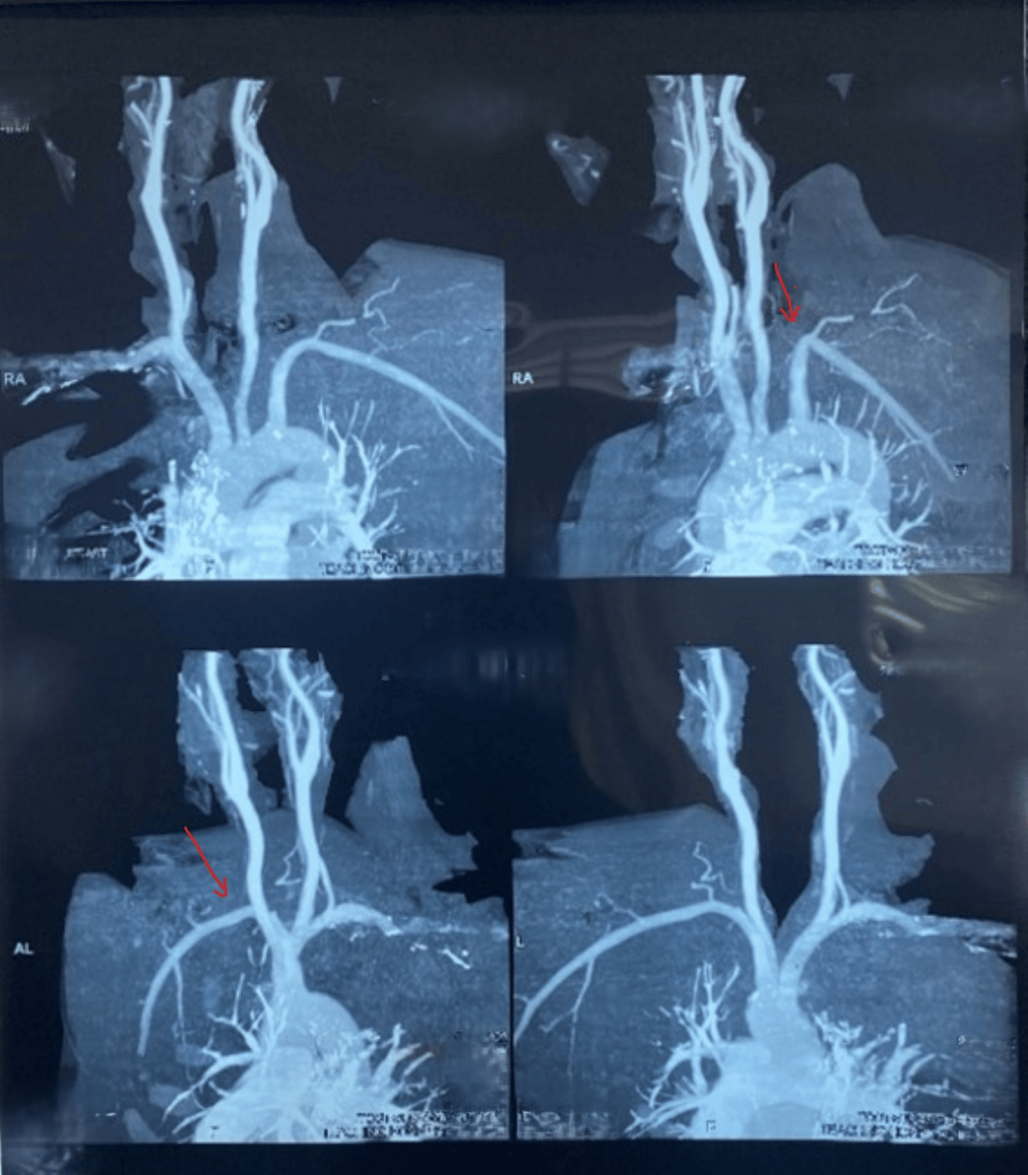
CT angiogram of the Aorta and major branches showing total occlusion of the pre-foraminal and proximal part of the foraminal segment of the left vertebral artery.

As the NCCT of the brain was normal, he was started on aspirin, clopidogrel, and atorvastatin. Due to sickling-related stroke, red cell exchange transfusion (target hemoglobin S <30%) and hydroxyurea were initiated. Ultrasound Doppler scan of the neck vessels showed retrograde flow in the left vertebral artery. CT angiogram revealed total occlusion of the pre-foraminal and proximal foraminal segments of the left vertebral artery (Figure 3).

His imbalance improved, but not the facial weakness and sensory loss. Regular red cell exchange transfusion was planned to keep the hemoglobin S level <30%. Family screening was performed for sickle cell disease.

## Discussion

This case highlights an uncommon yet significant complication of sickle cell trait: posterior circulation ischemic stroke due to vertebral artery occlusion. While sickle cell trait is often considered benign, it can predispose individuals to vaso-occlusion under specific conditions.

This patient, a previously healthy 41-year-old, had no major atherosclerotic risk factors except for smoking developed an acute ischemic stroke involving the left middle cerebellar peduncle, with prior lacunar infarction.

Sickle cell anemia is considered a risk factor for stroke. In conditions of mild hypoxemia, red blood cells sickle and sludge in arteries and veins, leading to thrombosis and infarction. Although red blood cells of heterozygous origin for sickle cell trait are more resistant to sickling, they too can sickle under conditions attainable in daily life and may also lead to infarction.

The percentage of hemoglobin S in sickle cell trait can vary from 25% to 45%. The higher the concentration of hemoglobin S, the higher the risk of sickling. However, a recent large study among African Americans suggested performing a more thorough clinical evaluation of a stroke patient with sickle cell trait presenting as stroke [2].

Some previous studies have suggested that the association of sickle cell trait and stroke is more than coincidental [1,4]. Our patient’s CT angiogram of the major branches of the aorta revealed total occlusion of the pre-foraminal and proximal part of the foraminal segment of the left vertebral artery. Vertebrobasilar insufficiency presents with “drop attacks,” but may also have various manifestations such as dizziness, diplopia, nystagmus, tinnitus, or even hearing loss. The two brief episodes of loss of consciousness before the presentation were likely due to transient vertebrobasilar insufficiency secondary to progressive occlusion. One episode occurred in a cold environment, and the other one during strenuous activity. Hence, the superimposed sickling could have resulted in further reduction of the flow during those episodes. The total occlusion can be attributed to chronic sickle vasculopathy. There have been cases reported on extracranial stenosis in sickle cell disease, which is rare [5]. No cases have been reported of sickle cell trait with extracranial carotid or vertebral arteriopathy.

## Conclusions

Screening for sickle cell disease/trait in young patients who present with stroke from disease clusters should be considered even in the absence of anemia or evidence of hemolysis. Sickle cell trait may also have complications similar to sickle cell disease, depending on the level of hemoglobin S. Ischemic stroke in sickle cell trait can be associated with extracranial carotid and vertebral arteriopathy. Further large-scale studies are required to determine the relationship between sickle cell trait and stroke.
